# Foreign Correspondence

**Published:** 1840-11-14

**Authors:** 


### MEDICAL EXAMINER.

DEVOTED TO MEDICINE, SURGERY, AND THE COLLATERAL SCIENCES.

No. 46.] PHILADELPHIA, SATURDAY, NOVEMBER 14, 1840. [Vol. III.

### FOREIGN CORRESPONDENCE.

To the Editors of the Medical Examiner.

### M. ORFILA ON SUICIDE FROM SUSPENSION.

It is now nearly two years since we published a short memoir on death from suspension,* in which we summarily presented the actual state of our knowledge on this delicate and important medico-legal subject. After offering an abstract of the opinions of the most distinguished writers on this question, we ventured in conclusion to express our own conviction of the great uncertainty that still remained attached to all the known physical signs, and the necessity of future investigation to enable us to declare with precision what are certain proofs of suspension during life. At the session of the *Academy of Medicine* on the 6th October, M. Orfila read an extended memoir on suspension, the substance of which we now propose to lay before our readers.

He commenced by stating his own impression of the great errors which prevailed on this subject in all medico-legal works, which were filled with contradictions, and vague assertions and litigations, not excepting even the most recent. Already the parchment furrow around the neck, considered by Mock as positive evidence, has been proved by Esquirol, Menierès, Devergie, and Casper of Berlin, to be of no value; as well as the seminal ejaculation, the congestive state of the genital organs, and the erection of the penis—these last being by no means constant phenomena, as proved by M. Orfila in his experimental essay on this subject on the 16th July, 1839. In a recent case M. Orfila was led to study with particular care the condition of the vertebral column in subjects dead from suspension; and on this point, as on others, he has found all previous writers in error.

On the 15th September, 1839, a man named Dauzet was found suspended by the neck in his stable, by a cord two yards long, the noose of which was passed over his shirt collar and that of his waistcoat. He was sitting on the ground, with his legs straightened out, at right angles with his body. A mass of lead fell from the fixed point of the cord upon the ground several inches. There was no injection around the neck. The mouth was filled with beans half digested. There were numerous ecchymosed spots upon the face. There was bloody extravasation in the scrotum and its tunics; but there were no traces of congestion in the genital organs, nor was there erection. The face was pale, and the pupils dilated. The shirt was spotted with blood around the genitals. The second cervical vertebra was, according to the report of the examiners, luxated to the left on the first, without rupture of the ligaments, or of the surrounding soft parts, and the finger could be passed between the left articular apophyses of the vertebræ. The sinuses of the dura mater were engorged, and the bladder contained urine in its natural state. There was no evidence on the body of any violence.

The two judicial examiners filed different reports. The one stated—

1. That Dauzet had died of asphyxia.
2. That suspension during life was not the cause of the asphyxia.
3. That the suspension occurred after death.
4. That art in a similar crime was insufficient, and could only be imagined from the contusion of the scrotum.

The other reporter, on the contrary, asserted—

1. That Dauzet had died of asphyxia.
2. That the asphyxia was the result of suspension during life.
3. That the ecchymoses and the bloody stains were far from proving that suspension had not taken place till after death.
4. That independent of the suspension, the cause of death would be unknown.

M. Orfila being called in for his opinion, thought that asphyxia was the cause of death. That, strictly speaking, it was possible to ad-

* Remarks on Suicide from Suspension. By Meredith Clymer, M D. Medical Examiner, vol. ii. p. 24.

mit that suspension had taken place during life, and was the result of suicide; but that this was in entire contradiction with the luxation of the second vertebra on the first, since all the circumstances forbid the idea of any resistance having been made.

The son of the deceased, and his wife, were tried for the crime, and convicted. After their condemnation they confessed the crime. They had choked the old Dauzet with his cap, at the same time turning the head several times on the trunk; and during the contest of the father against his son and his wife, the former had stamped violently on the abdomen with his feet, and bruized the genitals.

The object, then, of M. Orfila in the present memoir, was to dwell upon the value of the dislocations of the second vertebra on the first, and upon the other lesions of the vertebral column, as a means of diagnosis in cases of suspected suicide from suspension. He passed over rapidly the signs drawn from the state of the face, of the tongue, of the bloody mucus found in the mouth, of the rupture or not of the internal and middle coats of the carotid artery, of the marks of the cord, of the sperm found in the urethra, of the congestion of the genital organs, and of the erection of the penis. As to these last signs, M. O. expressed his conviction that they could exist as well in cases where suspension took place after death, if the suspension had been long; and that they were frequently wanting when suspension had occurred during life, particularly if the suspension was of short duration. Two cases of suicide by suspension, reported by M. Ollivier, (d'Angers,) proves this last assertion. In both instances the suspension lasted only a few minutes, and in neither was there any trace of injection, or of congestion, either in the scrotum or the penis, which was remarkably flaccid, and the corpora cavernosa very pale. In these two cases the cord had been passed above the thyroid cartilage, and the closure of the laryngeal opening had produced instantly asphyxia.

I. *Is it possible to produce a dislocation of the second and first vertebræ after death?*

On this point M. O. has made a series of experiments, which may be reduced to two series.

In the *first series* fourteen dead bodies were suspended, the cord being passed under the chin, the trunk very near the ground, and the legs drawn out, lay on the floor. The bodies were then subjected to violent and sudden jerks from above downwards, at the same time that there was powerful flexion and rotation of the head on the trunk. Once the odontoid process was fractured near its base, without displacement, because of the integrity of the ligaments. Once the body of the axis or second vertebra was fractured horizontally without displacement. In the remaining twelve no disturbance resulted from the same means. The body in which the odontoid was fractured was that of a slight built and emaciated woman, aged thirty-five; and the other was an old man of seventy-five years.

In the *second series*, six bodies were employed; these were hung at the distance of a yard from the ground. One or more strong men now threw themselves suddenly on their shoulders, the head being at the same time twisted in various directions. Accurate dissections revealed the existence of no lesion whatever.

The case of Dauzet is, however, at variance with these experiments. But it is necessary to remark that the reporters described this luxation as one without any rupture of the ligaments, or disturbance of the surrounding soft parts, and yet, nevertheless, that the finger could be introduced between the articular processes. How can we admit a luxation, with ability to introduce a finger between the articular processes, without rupture of the ligaments? On the other hand, how explain the two cases of fracture observed by M. Orfila? The age and condition of the body of these two subjects must be borne in mind, as well as this physiological circumstance, that the possible extent of the movement is very great in the direction of the articular surfaces. During life luxations ought to be less easy, for muscular contraction, then active, opposes a great resistance to communicated movements.

II. *Can we, by the preceding experiments, produce luxations or fractures of other parts of the vertebral column?*

In the *first series*, rupture of the yellow ligaments twice occurred, and, in one of the cases, there was the remarkable circumstance of extravasation of blood around the rent. Malle, in his experiments, was able to separate the spinous processes of the dorsal region, so as to place the finger between them. Once the body of a vertebra was fractured. Twice there was luxation of the articular processes of the dorsal vertebræ on one side. Six times some derangement in the bodies of the dorsal vertebræ, and in their articulations.

In the *second series* no result whatever was obtained. These great and varied lesions of the dorsal portion of the column are explained by the very limited motion it enjoys.

III. *Can luxation of the atlas on the axis take place in cases of execution by hanging?*

Many authors admit the affirmative, and the remarks and reasoning of Louis go to confirm this point. Nowhere has the celebrated secretary of the *Academy of Surgery* stated that he verified his facts by dissection, and the observation of J. L. Petit was not either confirmed by any autopsies.

On the other hand, Mackenzie and Monro, in England, dissected more than fifty criminals, and never found this lesion once. It is to be borne in mind, however, that the manner of hanging in England differs from that practised in France in the time of Louis.

IV. *Can dislocation of the second or the first vertebra occur in cases of self-destruction by suspension?*

Louis has answered in the negative. In all the autopsies of suicide by hanging, this lesion has never been found. Chaussier, in his lectures, mentioned a similar case, but he has not given the anatomical details. The ten other observations of a physician of Liege in proof of the possibility of this event, are too insignificant to merit serious attention.

V. *Can suspension during life, or after death, produce dislocation of the vertebral column below the second cervical vertebra?*

Mr. Orfila answers in the affirmative, and advances his experiments on the dead subject as proof. These disorders are favoured by the age and debility of the individuals.

VI. *Does there exist any characters drawn from the condition of the vertebral column which can enable us to determine whether the suspension was the effect of suicide or homicide?*

Contrary to the opinion of most writers, M. O. contends that the condition, exclusively, of the disorders of the vertebral column, is insufficieut to establish this question. The conclusions drawn by the profession are as follows:

1. Taken singly, no one of the signs given by writers enables us to determine whether the suspension took place before or after death. That M. Devergie was in error when he gave the five following characters as valuable: The asphyxia, the parchment ring around the neck, the fracture of the hyoid or thyroid cartilage, ejaculation, and section of the middle and internal coats of the carotid artery, a character given first by M. Amusat.

2. That when, with the signs of asphyxia, there is neither ecchymosis nor furrow around the neck, nor fracture, nor luxation, suspension can have taken place during life, but that it also, and which is most probable, could have occurred after death.

3. If there is an ecchymosis in the skin and cellular tissue around the parchment-like furrow, suspension during life is probable.

4. If there be rupture of the ligaments, and ecchymosis in the neck, signs of asphyxia, fracture of the vertebeæ, and no other lesions upon the body, the suspension could have equally occurred during life or not.

5. If with asphyxia, the body of any cervical vertebra is fractured, and there is absence of any appearance of violence, yet it is probably a case of homicide.

6. If there be luxation of the atlas upon the axis, we may safely affirm that suspension has followed death, unless there be caries of the vertebræ.

7. The proofs obtained at the place of the crime, and the collateral circumstances afforded us, are, after all, the best means of judging whether suspension occurred before or after death.*

After M. Orfila had finished reading his memoir, M. Dumeril mentioned the following case. Some years since, a few minutes after leaving the wards of his hospital at Rambouillet, his interne came to him in haste, telling him that the patient he (M. D.) had just quitted, had hung himself. The next moment the man was cut down. He was, however, dead. The next day, on examining him, he found rupture of the odontoid ligaments, luxation of the odontoid process, which had passed under the transverse ligament, and compressed the spinal marrow. The autopsy was made by M. D. himself.

* Vide Medical Examiner, vol. ii. p. 26, 1839.

M. Velpeau said that he did not consider it correct to state that muscular contraction rendered luxation more difficult during life, for it is well known that this force, when once the bones are placed in a certain direction, is itself added to the external causes, and favours the production of dislocations. M. Gimelle related a case similar to that of M. Dumeril, in which no lesion whatever was found in the vertebral column. There was ejaculation, but no congestion in the genital organs, or the slightest erection.

M. Dupuy stated that he had made a number of experiments upon horses, and the result was to diminish the value of the ecchymosis as a sign. He injected into the veins of horses, blood mixed with cerebral matter, and then killed them. On opening them, some hours afterwards, he found large patches of ecchymosis in the lungs. In others which were examined a half an hour after the injection, he found only slight ecchymosed spots, and, on continuing his researches in other organs, he saw these ecchymosed specs increase gradually, under his eyes, until they reached the size of those examined seven hours after death.

Paris, 13*th October*, 1840.

